# Ecological driving on multiphase trajectories and multiobjective optimization for autonomous electric vehicle platoon

**DOI:** 10.1038/s41598-022-09156-2

**Published:** 2022-03-25

**Authors:** Tang Xiaofeng

**Affiliations:** School of Mechanical Engineering, Yangzhou University, Yangzhou, Jiangsu China

**Keywords:** Mechanical engineering, Electrical and electronic engineering

## Abstract

Autonomous electric vehicles promise to improve traffic safety, increase fuel efficiency and reduce congestion in future intelligent transportation systems. Ecological driving characteristics are first studied to concentrate on energy consumption, the ability to quickly pass its destination, etc. of autonomous electric vehicle plans (AEVPs) to maximize total energy efficiency benefits. To realize this goal, an optimal control model is developed to provide ecological driving suggestions to AEVPs. The Radau pseudospectral method (RPM) is adopted to put the optimal control model into nonlinear programs (NLP), and multiobjective optimization, including safety, economy and fast mobility, is considered, which conditions and constraints such as vehicle dynamics, traffic rules, and energy consumption. To enhance optimal model applicability, two ecological driving procedures are proposed. One procedure is that two-phase trajectory optimization and ecological driving states, such as velocity and acceleration, for the leading vehicle are developed according to RPM characteristics, while the other provides a set of targeted driving states to the following vehicles. The objective of the procedure is to minimize the total energy consumption of AEVPs, while travel comfort and safety are integrated into the schematization by optimization functions. Numerical experiments illustrate significance when ecological driving strategy for AEVPs considers energy consumption characteristics, thereby ensuring total energy consumption efficiency for AEVPs.

## Introduction

### Background and motivation

The automotive industry has developed electric vehicles to reduce the dependency on petroleum fuels, improve energy efficiency and promote sustainable transportation. Many governments have plans to accelerate the market shares of electric vehicles (EVs); therefore, EVs have experienced huge market penetration over the past decade. The U.S. EIA projects that over 30 million (24%), passenger cars on U.S. roads will be electricity powered by 2040^1^. EVs will be common transportation in future.

Ecological electric vehicles (EEVs) are a new concept that adapts the driving strategy to an energy-aware anticipative driving strategy and have high potential to improve the safety and efficiency of the transportation network^2^. EEVs mainly mean that energy consumption needs to be considered to cover travel activities due to battery technology limitations, combined with vehicle powertrains, vehicle dynamics, tire models, etc. Meanwhile, surrounding vehicle states and road information can be collected by connecting vehicle technologies to determine innovative driving state control and trajectory planning. Edwin proposed an approach to estimate the potential of eco-driving for reducing energy consumption^3^. Maria introduced the Pontryagin maximum principle to optimize the calculated battery consumption, and connected vehicle technology was used to transmit with other vehicles^4^. Hu proposed an optimal controller by using emerging connected technology to minimize the fuel efficiency for hybrid electric vehicles, including road topography and dynamic speed limits^5^. Therefore, ecological driving is an effective integrated strategy that includes vehicle dynamics control, energy consumption calculation, vehicle driving state collection and connected vehicle technology. In particular, for electric vehicles, regenerative braking can recharge a portion of energy to the battery during deceleration. Existing study results demonstrate that regenerative braking could increase the EV driving range by 8–25%^6^.

A platoon is another energy-efficient vehicle driving control solution that can save battery fuel. Sun shows that the average reduction in fuel consumption for platoons can reach up to 10%^7^. Vehicle platoons are mainly used to control a suitable safety distance to reduce air resistance and energy consumption by installing sensors such as radar, lidar or machine vision. Recently, connected vehicle technologies have been applied to platoons that can enable vehicles to drive a much closer distance upon information obtained from neighboring vehicles. Many studies mainly design optimal control models to obtain speed advisories for minimizing fuel consumption. Gong developed cooperative platoon control for a platoon mixed with connected and autonomous vehicles and human-driven vehicles by using the model predictive control optimal method^8^. Jia builds a cooperative driving model by using connected vehicle technologies for studying time-delay communication^9^. Luo introduced a mixed-integer linear program to minimize the total fuel consumed with multiple speed options^10^. Li proposed a Lyapunov technique to study trajectory tracking control for connected vehicle platoons by considering the position error and velocity difference^11^. The above studies generally concentrate on algorithm theories, optimal models and fuel energy consumption for human-driving vehicle platoons. However, fuel consumption calculations for vehicle platoons should be taken into consideration; in combination with vehicle dynamics and road information, the driving states of leading vehicles are usually assumed or ignored so that the total fuel energy consumption cannot be accurately calculated. In fact, regarding energy-efficient vehicle platoons, the driving states of leading vehicles are important, especially for electric vehicle platoons under congested traffic conditions; therefore, multiobjective optimization, including safety, economic and fast mobility, should be calculated in ecological driving studies. Lorenzo introduced nonlinear model predictive control to develop an ecological driving of electric vehicles to minimize energy consumption^12^. Makoto proposed adaptive neural networks to design electric vehicle platoons^13^. Guo introduced an adaptive backstepping sliding mode of a high-level law to study the coordinated control method for autonomous electric vehicle platoons^14^. Several studies have developed innovative control algorithms, stability analysis and parts of fuel energy consumption. However, the total fuel energy consumption minimization of electric vehicle platoons should be researched due to the energy consumption characteristics of multivehicle platoons.

### Autonomous electric vehicle

Recently, the majority of EV tasks have mainly focused on battery technology limitations and charging infrastructure problems, while the driving efficiency of EVs should be studied in an intelligent transportation system environment. Autonomous electric vehicles, combined with connectivity, are considered a major issue supporting a more efficient and clearer transportation system. Gao thinks that adopting connected and automated vehicle technologies can promote energy efficiency via eco-driving functions in optimizing EV performance effectively^6^. Riccardo proposed a mixed-integer linear program methodology for the optimization of charging with a vehicle-to-grid for a shared autonomous electric vehicle^15^. Chen developed a passive fault-tolerant path following the control method of an autonomous distributed electric vehicle when a vehicle steering system is fault^16^. Yi proposed a data-driven method to formulate a grid stochastic energy consumption model of electric vehicles^17^. Zhang developed a novel tracking control method of intelligent electric vehicles to realize path tracking based on lane detection and sliding mode control methods^18^. Victor designs a static decision model to involve autonomous electrical vehicles^19^. Li proposed a potential field method to achieve the trajectory control of autonomous electric vehicles with in-wheel motors^20^. Doan proposed the Pontryagin maximum principle for the optimal allocation of wireless power transfer systems^21^. The above studies mainly focus on performance optimization, algorithm theories, path-following methods and charging methods, etc., while fuel consumption energy has not been considered in the process of trajectory planning for autonomous electric vehicles. In fact, energy consumption is a vital factor for autonomous electric vehicles because limited energy should be used to ensure that autonomous electric vehicles arrive at the terminal position.

Another energy-efficient trajectory plan for autonomous electric vehicles is platooning, which shows good potential for fuel economy. As autonomous electric vehicle platoons (AEVPs) travel on roads, different energy consumption characteristics and constraints for different vehicles can be allocated through new technologies, such as vehicle-to-vehicle (V2 V) and vehicle-to-infrastructure (V2I) communications or algorithm innovation. In this way, if trajectory planning and ecological driving states of leading vehicles can be effectively designed, the following vehicles can realize safety, energy-efficient trajectory tracking and ecological driving states. In fact, many studies mostly focus on the driving states of the following vehicles, and the driving states of the leading vehicle are ignored so that the predictive driving states of the leading vehicle cannot be adjusted according to the specific driving environment. Therefore, the total energy consumption for AEVPs could not be truly realized. Choi proposes RFID tags to supply lines and estimate the position of vehicles, and connecting technologies are used to calculate distances between vehicles^22^. Zhao developed V2 V communication to realize autonomous electric vehicle platoons, and thus vehicle interruptions were successfully designed^23^. He develops an optimal control model as a foundation to provide ecological driving to mixed gasoline vehicles and EVs, and speed advisory and acceleration-based advisory strategies are used to study energy consumption^1^. Bian studied a multiple-predecessor following strategy to reduce time headway via vehicle-to-vehicle (V2 V) communication, and a constant time headway policy was proposed for general communication topologies^24^. Qi designed a cooperative system in which vehicles and infrastructure are tightly integrated by using wireless communications^25^. In addition, the impact of driving behavior, vehicle dynamics and data-driven energy consumption are also integrated into the framework. The above studies on AEVPs have been chiefly embodied in V2 V systems, optimal control models and algorithm innovation. In fact, ecological driving for AEVPs is a local minimum problem due to constraints imposed by neighboring vehicles. Therefore, to ensure the minimum total energy consumption of AEVPs, the ecological driving states of leading vehicles and following vehicles should be jointly studied. Especially importantly, ecological trajectories of leading vehicles should be designed to ensure effective vehicle tracking according to specific traffic environments.

### Motion planning algorithm

Motion planning aims to plan motion trajectories in a short future horizon to find collision-free trajectories under static and dynamic obstacles. The majority of existing studies in motion planning algorithms of autonomous vehicles are found in the robotics field. There exist three category methods. First, the sampling-based method means that the state space is discretized or randomly sampled in lattices, such as the A* graph and RRT algorithm. Second, the decomposed method means that the planning problem is decomposed into two easier subproblems. Third, the motion planning problem is transferred into a mathematical programming-based method, such as model predictive control^26^. Hu introduced an improved artificial potential method for path planning considering vehicle velocity to prevent unnecessary lane changing behavior^27^. Song proposed an improved A* graph, and a new path smoothing process with three types of path smoothers was developed to improve path performance^28^. Mohamed introduced the RRT algorithm to split planning into two efficient phases to reduce its computational time combined with the robustness of parametric vector valued splines. Other methods of motion planning have also been developed in recent years^29^. Wu developed differential dynamic programming for a novel path planner combined with kinematic feasibility^30^. Li proposed a novel integrated local trajectory planning and tracking control framework for motion planning of autonomous vehicles^31^. The above studies are mainly focused on motion planning algorithms to realize specific driving behaviors. However, these algorithms only generate instantaneous commands, and the generated trajectories are not smooth or ignore vehicle dynamics and traffic rule safety constraints. Guo introduced a model predictive control method to design path-following schemes for autonomous vehicles, and a differential evolution algorithm was adopted^32^. Makoto developed nonlinear model predictive control for autonomous vehicle motion planning, which can solve nonlinear vehicle dynamics and environmental conditions^33^. Bian presents a systematic approach to connected vehicles at unsignalized intersections without global coordination, and a distributed observation algorithm is introduced to achieve trajectory planning^34^. Indeed, safety requirements for autonomous vehicles are to ensure vehicle dynamics safety, especially when vehicles are at high speeds. Meanwhile, tire mechanics and rollover should be taken into consideration. However, autonomous vehicles are complicated engineering artifacts, so many problems in motion planning cannot be solved by simply relying on model predictive control methods. For example, multiphase trajectories for autonomous vehicles should be planned according to specific constraints for specific traffic scenes, the immersed local optimal problem should be solved to realize global optimization, and terminal states of autonomous vehicles should be defined according to specific road traffic environments. Sometimes we want to compute a trajectory from its current position to the destination as fast or as energy efficient as possible^35^. Therefore, to achieve the requirements of autonomous vehicles, another trajectory planning method should be developed. The pseudospectral method was applied in the field of aerosols in the early stage. Few papers have been published using the pseudospectral method to solve motion planning for autonomous vehicles. Michael introduced a pseudospectral optimal control algorithm for motion planning of small unmanned ground vehicles^36^. The pseudospectral algorithm is a direct approach in which numerical optimal control techniques are used to solve the motion planning of robots, and the abovementioned problems can be solved by the pseudospectral method. The pseudospectral algorithm is an effective optimal control method for autonomous vehicles.

### Research contributions

This study aims to address the ecological driving on multiphase trajectories and multiobjective optimization for AEVPs by using the pseudospectral method and V2V communication technologies to minimize the total fuel consumption of AEVPs. Ecological driving means that the constraints of vehicle dynamics, traffic rules, energy consumption minimization, and the ability to quickly pass trajectories of electric vehicles should be taken into consideration when trajectory planning and driving states of AEVPs are designed in this paper. The first strategy shows that the pseudospectral method is used to design ecological driving on trajectory planning and driving states of the leading electric vehicle to ensure ecological driving of the leading vehicle. The second strategy shows that the following electric vehicles should track the planned multistage trajectories, and the driving states of the leading vehicle can be transmitted to following electric vehicles by V2 V communication technologies under the conditions of ensuring the minimum total platoon energy consumption. In addition, a multiobjective optimal model including safety, economic and fast mobility mainly involves two parts: the minimum of total energy consumption and the ability to quickly pass its destination to realize ecological autonomous driving.

The remainder of the paper is organized as follows. “System models for ecological drving of AEVPs” Section provides system models including longitudinal vehicle kinematics models, vehicle dynamics models and platoon vehicle dynamics models for AEVPs. “Pseudospectral algorithm for solving system models” Section presents a pseudospectral method that is suitable for platoons with an automated leader and following electric vehicles. “Optimal control model formulation” Section proposes an optimal model that will be used to solve ecological driving states for AEVPs. “Numerical experiments” Section provides some experiments and concluding comments.

## System models for ecological drving of AEVPs

Autonomous electric vehicles are researched in this paper to improve energy efficiency, promote sustainable transportation and realize green mobility, especially as ecological driving on AEVPs and reducing overall fuel consumption are helpful to realize high-quality green mobility. Autonomous electric vehicle applications are mostly focused on vehicle system models to improve efficiency through optimal powertrain operations. Vehicle dynamics models, longitudinal vehicle kinematics models, platoon dynamics models and energy consumption models are involved in system models. Longitudinal vehicle kinematics models are used to calculate the energy consumption of AEVPs, and vehicle dynamics models are used to design motion planning and driving states for the leading vehicle according to specific traffic scenes. System models are crucial to ecological driving for AEVPs.

### Longitudinal vehicle kinematics models

Longitudinal vehicle kinematics models of AEVs include traction force, ground resistance and wind resistance. Newton’s second law is adopted to build longitudinal models, as follows:

1 $$\left\{ \begin{aligned}& \frac{{ds_{i} }}{dt} = v_{i} \hfill \\ &\frac{{dv_{i} }}{dt} = \frac{{F_{{t_{i} }} - \sum {F_{{res_{i} }} } }}{{m_{{e_{i} }} }} \hfill \\ \end{aligned} \right.$$

In the above equation, $i = 1,2, \ldots ,N$ represents every vehicle in the platoon, $s_{i}$ represents the motion of the vehicle at the position, and velocity $v_{i}$ is assumed to be a point mass at the center of gravity. $F_{{res_{i} }}$ represents the total resistive forces, and $m_{{e_{i} }}$ is the mass of EV. $F_{{t_{i} }}$ is traction force.

Electric vehicle is assumed to front-wheel drive. The traction force can be expressed as:

2 $$F_{{t_{i} }} = \frac{{i_{g} \eta_{t} \tau_{t} }}{{r_{d} }}$$

where $r_{d}$ is the effective radius of the wheel, $i_{g}$ is the transmission ratio, $\tau_{t}$ is the powertrain torque output, and $\eta_{t}$ is the total mechanical transmission efficiency.

The total resistive forces are aerodynamic drag ($F_{{w_{i} }}$), grading resistance ($F_{{g_{i} }}$), and tire rolling resistance ($F_{{r_{i} }}$). The $F_{{res_{i} }}$ can be expressed as:

3 $$\sum {F_{{res_{i} }} } = F_{{w_{i} }} + F_{{g_{i} }} + F_{{r_{i} }}$$

Aerodynamic drag is related to vehicle velocity as follows:

4 $$F_{{w_{i} }} = \frac{1}{2}\rho_{a} A_{f} C_{D} v_{i}^{2}$$

where $\rho_{a}$ is the air density, $A_{f}$ is the vehicle frontal area, and $C_{D}$ is the aerodynamic drag coefficient.

Grading force can be expressed as:

5 $$F_{{g_{i} }} = m \cdot g \cdot \sin \theta$$

Rolling resistance can be expressed as:

6 $$F_{{r_{i} }} = \mu \cdot m_{{e_{i} }} \cdot g \cdot \cos \left( \theta \right)$$

where $$g = 9.8{\raise0.7ex\hbox{$m$} \!\mathord{\left/ {\vphantom {m {s^{2} }}}\right.\kern-\nulldelimiterspace} \!\lower0.7ex\hbox{${s^{2} }$}}$$, $$\mu$$ is rolling resistance coefficient.

### Energy consumption model

The driving of electric vehicles depends on motors; therefore, it is necessary to establish a mechanical power and energy consumption model of the motor, and vehicle braking energy recovery is also considered. The energy consumption of electric vehicles depends on speed characteristics, acceleration characteristics, road geometry characteristics and road traffic conditions. The regenerative braking system can recharge a portion of energy to the battery. Therefore, the relation between the mechanical power of the motor and energy can be shown as:

7 $$P_{{M_{{\text{i}}} }} = w_{{M_{{\text{i}}} }} \cdot T_{{M_{{\text{i}}} }}$$

The driving power can be described as:

8 $$P_{{M,e_{{\text{i}}} }} = \left\{ \begin{aligned} & \frac{{P_{{M_{i} }} }}{{\eta_{{M_{i} }} }},P_{{M_{i} }} \ge 0 \, \hfill \\ & P_{{M_{i} }} \cdot \eta_{{M_{i} }} ,P_{{M_{i} }} < 0 \, \hfill \\ & \eta_{{M_{i} }} = f_{{1}} \left( {w_{{M_{i} }} ,T_{{M_{i} }} } \right) \hfill \\ \end{aligned} \right.$$

where $\eta_{{M_{i} }}$ is the motor transmission efficiency, $P_{{M_{i} }}$ is the motor mechanical power, $w_{{M_{i} }}$ is the energy required for mileage, and $T_{{M_{i} }}$ is the motor torque. When the vehicle drives, the consumed power can be calculated in the first equation in Eq. (8). When the vehicle is regenerative braking power in brake mode, the second equation in Eq. (8) can be used.

Through the above formulation, it is known that the motor will consume part of the energy, and the function of regenerative braking energy recovery should be obtained when optimization models are built in the paper. The braking energy can be recovered by 60% when the braking force is small. In contrast, the braking energy can be recovered by 30%-40% when the braking force is large in this paper.

### Vehicle dynamics model

**Figure 1:**
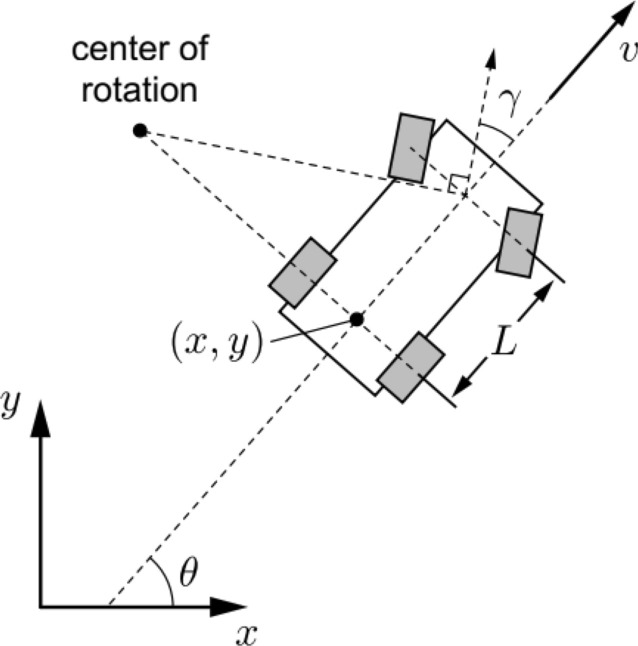
Vehicle Dynamics Model.

Vehicle dynamics models are increasingly applied to realize ecological driving for AEVs and are mainly used to design motion planning and driving states for the leading vehicle according to specific traffic scenes. The following vehicles can track the planned trajectory and adjust its driving state by V2 V communication technologies. Vehicle dynamics can be shown in Fig. 1^36^.

According to Fig. 1, the vehicle dynamics model is expressed as follows:

9 $$X = \left[ \begin{array}{l} {\dot x}\\ {\dot y}\\ {\dot \theta }\\ {\dot v}\\ {\dot \gamma } \end{array} \right] = \left[ \begin{array}{l} v\cos \theta \\ v\sin \theta \\ \frac{v}{L}\tan \gamma \\ a\\ w \end{array} \right]$$

where $x,y,\theta$ is the vehicle position and direction, $v,L$ is the vehicle velocity and distance, $a,\gamma$ is the acceleration and steering angle ratio, $w$ is the yaw angle, and $u = [a,w]^{T}$ are control variables that ensure system continuity.

### Platoon dynamics model

The objective of platoon control is to track the driving states of the leading vehicle and maintain a desired distance between consecutive vehicles, as follows:

10 $$\left\{ \begin{aligned}& \mathop {\lim }\limits_{t \to \infty } \left\| {v_{i} \left( t \right) - v_{0} \left( t \right)} \right\| = 0 \hfill \\ & \mathop {\lim }\limits_{t \to \infty } \left\| {s_{i - 1} \left( t \right) - s_{i} \left( t \right) - d_{0} } \right\| = 0 \hfill \\ \end{aligned} \right.$$

where $d_{0}$ is the desired space between vehicles. To ensure safe driving for AEVPs, the relative vehicle velocity and relative distance are designed, and a desired space between vehicles is also designed to arrive at the goal.

The objective of this study is to form ecological driving for AEVPs, so the intelligent driver model (IDM)^37^ is adopted as a vehicle-following model to research the speed and acceleration of vehicle platoons. The IDM formulates acceleration rate as follows:

11 $$a_{i} \left( t \right) = a_{i - i} \left[ {1 - \left( {\frac{{v_{i} \left( t \right)}}{{v_{i}^{d} }}} \right)^{4} - \left( {\frac{{\Delta {\rm H}_{i} \left( {v_{i} \left( t \right),\Delta v_{i} \left( t \right)} \right)}}{{\Delta {\rm H}_{i} \left( t \right)}}} \right)^{2} } \right]$$

where $v_{i}^{d}$ represents the desired velocity of vehicle $i$, $a_{i - i}$ is the comfortable acceleration rate of vehicle $i$, $a_{i - i} { = 4}m/s^{2}$ is used in this problem, $\Delta v_{i} \left( t \right)$ is the speed difference between vehicles, $\Delta {\rm H}_{i} \left( {v_{i} \left( t \right),\Delta v_{i} \left( t \right)} \right)$ is the expected speed of the vehicle in free traffic flow, and $\Delta {\rm H}_{i} \left( t \right)$ is the gap between the following vehicle and the leading vehicle.

The desired distance between vehicles is defined as follows:

12 $$\Delta x_{i} \left( {v_{i} \left( t \right),\Delta v_{i} \left( t \right)} \right) = s_{0} + \max \left( {v_{i} \left( t \right) \cdot t + \frac{{v_{i} \left( t \right) \cdot \Delta v_{i} \left( t \right)}}{{2\sqrt {a_{i - i} \cdot a_{i}^{d} } }},0} \right)i = 2,...,N$$

where $s_{0}$ denotes the jam distance, $t$ is the safety time headway, and $a_{i}^{d}$ is the desired deceleration rate.

## Pseudospectral algorithm for solving system models

### Pseudospectral algorithm

Pseudospectral algorithms are direct methods in the numerical method field and can also be named global optimization algorithms, usually Lagrange interpolation is used to put a series of optimal problems into discretization and realize the global solution, while the real optimum sometimes cannot be obtained. Generally, direct methods transcribe the continuous optimal control problem to nonlinear programs (NLPs) based on the discretization method^38^. Pseudospectral algorithms can be categorized into the Gauss pseudospectral method (GPM), Legendre pseudospectral method (LPM) and Radau pseudospectral method (RPM). Pseudospectral methods have attracted much attention because of their exponential convergence rates for problems with smooth and well-behaved solutions^39^. In RPM, the Legendre–Gauss-Radau (LGR) collocation method is adopted to discretize the optimal control problem. In this study, RPM is proposed to develop ecological driving for AEVPs because it has the advantage of exponential convergence, and state interpolation, such as terminal points and constraints, is included. It also has the advantages of fast convergence, lower sensitivity to initial values and multistage trajectory optimization and can obtain higher accuracy results with less computational cost. Accordingly, an optimal control model based on RPM is studied to minimize the total energy consumption of AEVPs to realize ecological driving. In addition, for a platoon, the optimal model needs energy-optimal acceleration. Therefore, putting nonlinear optimal control problems into a nonlinear programming problem (NLP) to realize total energy consumption minimization of AEVPs is an important method. The specific procedure using RPM is as follows:

First, time regions. To apply the RPM algorithm, the time region to achieve the optimal control problem is $\tau \in [ - 1,1]$. Therefore, the system optimization problem of time region $\left[ {t_{0} ,t_{f} } \right]$ should be transmitted into $[ - 1,1]$, and time variable t can be defined as follows:

13 $$t = \frac{{t_{0} + t_{f} }}{2} - \frac{{t_{0} - t_{f} }}{2}\tau$$

If the ecological driving process of AEVPs has been divided into K phases that have consisted of K mesh intervals, the time region in every phase should be transmitted into $[ - 1,1]$.

Second, discretion state variables and control variables. The RPM principle shows that Lagrange interpolation is used to put a series of optimal problems into discretization by building Lagrange polynomials to approximate states $x\left( \pi \right)$ and $u\left( \tau \right)$, especially in K phases of RPM. Suppose that the time variable has been divided into K subregions: $S_{k} = [t_{k - 1} ,t_{k} ]$,$\left( {k = 1,2, \ldots ,K} \right)$. The state variable and control variable in mesh space $k \in [1,2, \ldots ,K]$ are approximated as follows:

14 $$\left\{ \begin{gathered} x^{k} (\tau ) \approx X^{k} (\tau ) = \sum\limits_{i = 1}^{{N_{k} + 1}} {L^{k}_{i} (\tau )} X_{i}^{k} \hfill \\ u^{k} (\tau ) \approx U^{k} (\tau ) = \sum\limits_{i = 1}^{{N_{k} + 1}} {L^{k}_{i} (\tau )U_{i}^{k} } \hfill \\ \end{gathered} \right.$$

where $L^{k}_{i} (\tau ) = \prod\nolimits_{j = 1,j \ne i}^{{N_{k} + 1}} {\left[ {\frac{{\tau - \tau_{j}^{k} }}{{\tau_{i} - \tau_{j}^{k} }}} \right]}$, $\tau \in \left[ { - 1,1} \right]$, $L^{k}_{i} (\tau )$, $i = 1,2, \ldots ,N_{k} + 1$ is the interpolation basis function, and $j = 1,2, \ldots ,N_{k}$ is the collocation point in $k \in [1,2, \ldots ,K]$.

Third, Dynamical differential equation conversion. Equation (14) is derived and transformed into matrices as follows:

15 $$\sum\limits_{i = 0}^{K} {D_{ki} \cdot X_{{\text{i}}} { - }\frac{{t_{f} - t_{0} }}{{2}}f_{{2}} \left( {X_{k} ,U_{k} ,\tau_{k} ;t_{0} ,t_{f} } \right) = 0}$$

where $$D_{ki} { = }\mathop {{\dot L}{}_{i}}\nolimits \left( {\tau_{k} } \right) = \sum\nolimits_{l = 0,l \ne i}^{K} {\frac{{\prod\limits_{j = 0,j \ne i,l}^{K} {\tau_{k} - \tau_{j} } }}{{\prod\limits_{j = 0,j \ne i}^{K} {\tau_{i} - \tau_{j} } }}}$$, $$k = 1,2, \ldots ,K$$, $$i = 0,1, \ldots ,K$$.

Forth, Objective function. Through the above three steps, the optimization function can be calculated as follows:

16 $$J = M\left( {X_{1}^{1} ,t_{0} ,X_{{N_{k} + 1}}^{K} ,t_{K} } \right) + \sum\limits_{k = 1}^{K} {\sum\limits_{l = 1}^{{N_{k} }} {\left( {\frac{{t_{k} - t_{k - 1} }}{2}} \right)} } \mu_{i}^{k} L\left( {X_{l}^{k} ,U_{l}^{k} ,\tau_{l}^{k} ;t_{k - 1} ,t_{k} } \right)$$

where $\mu_{i}$ is integration weight of Gauss-Radau.

### Boundary conditions and constraints

Ecological driving for AEVPs needs to take into consideration boundary conditions to make AEVPs safely, effectively and ecologically track trajectory and driving states. A multiobjective optimization model also needs to consider the constraints and conditions. The boundary conditions and constraints include the driving states of the leading vehicle and following vehicles, initial boundary conditions and terminal boundary conditions. Initial boundary conditions are initial state variables at the beginning of vehicles, and terminal boundary conditions are the conditions that need to be satisfied at the end of trajectory. Two phase trajectories for AEVPs are studied, and multiobjective state constraints are set up according to specific traffic scenes.

In the first phase, the trajectory and driving states of the conditions and constraints of the leading vehicle should be set up as follows:

The initial states of the leading vehicle at the beginning include the vehicle position, velocity, heading angle, and front wheel angle as follows:

17 $$X_{{{10}}} { = }\left\{ \begin{aligned} &x_{{{1}0}} = x_{0} ,y_{10} = y_{0} , \hfill \\& \theta_{10} = \theta_{0} ,v_{10} = v_{0} ,\gamma_{10} = \gamma_{0} \hfill \\ \end{aligned} \right\}$$

The terminal states of the leading vehicle at the end in the first phase can be set up as follows:

18 $$Y_{{{\text{1f}}}} { = }\left\{ \begin{aligned} &x_{1f} = x_{f} ,y_{1f} = y_{f} , \hfill \\ & \theta_{1f} = \theta_{f} ,v_{1f} = v_{f} ,\gamma_{1f} = \gamma_{f} \hfill \\ \end{aligned} \right\}$$

The leading vehicle should obey traffic rules, and driving states should be restrained in specific ranges to ensure AEVP ecological driving according to specific traffic scenes. Therefore, the state constraints for the leading vehicle are:

19 $$\begin{aligned} & x \in \left[ {x_{1\min } ,x_{1\max } } \right],y \in \left[ {y_{1\min } ,y_{1\max } } \right],\theta \in \left[ {\theta_{1\min } ,\theta_{1\max } } \right], \hfill \\ & v \in \left[ {v_{1\min } ,v_{1\max } } \right],\gamma \in \left[ {\gamma_{1\min } ,\gamma_{1\max } } \right] \hfill \\ \end{aligned}$$

Control variables should obey vehicle dynamics, and their constraints are:

20 $$a \in \left[ {a_{1\min } ,a_{1\max } } \right],w \in \left[ {w_{1\min } ,w_{1\max } } \right]$$

In the second phase, the initial states of the leading vehicle at the beginning are equal to the terminal states of the leading vehicle in the first phase according to the principle of RPM:

21 $$X_{20} = Y_{{{\text{1f}}}}$$

The terminal states of the leading vehicle at the end in the second phase can be set up as follows:

22 $$Y_{{{\text{2f}}}} { = }\left\{ \begin{aligned} & x_{2f} = x_{f - f} ,y_{2f} = y_{f - f} , \hfill \\ & \theta_{2f} = \theta_{f - f} ,v_{2f} = v_{f - f} ,\gamma_{2f} = \gamma_{f - f} \hfill \\ \end{aligned} \right\}$$

The state constraints of the leading vehicle in the second phase are:

23 $$\begin{aligned} & x \in \left[ {x_{2\min } ,x_{2\max } } \right],y \in \left[ {y_{2\min } ,y_{2\max } } \right],\theta \in \left[ {\theta_{2\min } ,\theta_{2\max } } \right], \hfill \\ & v \in \left[ {v_{2\min } ,v_{2\max } } \right],\gamma \in \left[ {\gamma_{2\min } ,\gamma_{2\max } } \right] \hfill \\ \end{aligned}$$

Initial states of following vehicle $i$ at the beginning include distances between vehicles, and velocities should be set up as follows:

24 $$s_{0i} = s_{0} ,v_{0i} = v_{0}$$

The terminal states of vehicle $i$ at the end are:

25 $$s_{fi} = s_{f} ,v_{fi} = v_{f}$$

The constraints of driving states for following vehicle $i$ are:

26 $$s_{i} \in \left[ {s_{i\min } ,s_{i\max } } \right],v_{i} \in \left[ {v_{i\min } ,v_{i\max } } \right]$$

## Optimal control model formulation

The function of the proposed optimal control model is to minimize the total energy consumption of AEVPs to realize ecological driving and achieve automatic driving. Therefore, the objective of the optimal model is to minimize total energy consumption, comfort, safety and the ability to quickly pass its destination, which shows exponential convergence and high efficiency. Multi-objective optimization could arrive at a good effect, for example, when total energy consumption is calculated, vehicle safety is needed to considered to ensure the suitable speed planning, and the vehicle comfort is also considered, because the relation between velocity planning and vehicle comfort is demanded to balance. Many scholars are studied optimal relation such as optimal control for a semi-autonomous ecological driver assistance system^2^, energy efficient speed planning for optimal control problem^40^.Meanwhile, nonlinear optimal control problems are converted into NLP to solve the abovementioned objectives. For a platoon approaching road section that is prone to traffic accidents, the optimal control model needs energy-optimal acceleration, as follows:

27 $$\mathop {\min }\limits_{a\left( t \right),t} \xi = \mho_{1} \cdot \xi_{{1}} { + }\mho_{{2}} \cdot \xi_{{2}} { = }\mho_{1} \cdot \sum\limits_{i} {\int_{0}^{{t_{f} }} {E_{i} \left( {v_{i} \left( t \right),a_{i} \left( t \right)} \right)} } dt + \mho_{2} \cdot t_{f}$$

Subject to:

28 $$\begin{aligned} \mathop {{\dot x}_{i} }\limits \left( t \right) &= v_{i} \left( t \right),0 \le t \le T,i = 1,2, \ldots ,N \hfill \\ \mathop {{\dot v}_{i} }\limits \left( t \right) &= a_{i} \left( t \right),0 \le t \le T,i = 1,2, \ldots ,N \hfill \\ \end{aligned}$$

29 $$a_{i} \left( t \right) = f\left( {\Delta x_{i} \left( {t - T_{1} } \right),v_{i} \left( {t - T_{1} } \right),\Delta v{}_{i}\left( {t - T_{1} } \right)} \right)$$

Combined with Eqs. (17), (18), (21), (22), (24), and (25) and constraints Eqs. (19), (23), and (26), the optimal control model can be transferred to the NLP problem.

In the above optimal model, Eq. (27) is the total energy consumption minimizing model of AEVPs traveling from the location to the next position and time to quickly pass its destination. The minimum target state considers instantaneous energy consumption calculations and the ability to quickly pass the traffic capacity of vehicles. Vehicle acceleration as a control variable should be set to reasonable optimal values to realize the energy consumption minimum, so the weight for instantaneous energy consumption should be set as a suitable value.

$E_{i} \left( {v_{i} \left( t \right),a_{i} \left( t \right)} \right)$ represents the instantaneous energy consumption rate of vehicle $i = 1,2, \ldots ,N$, which is a function of velocity $v_{i} \left( t \right)$ and acceleration $a_{i} \left( t \right)$ at time t. The ecological driving for AEVPs mainly optimizes acceleration. $t_{f}$ means that the vehicle can be brought from the current position to its destination as fast as possible. $\xi_{{1}}$ is the instantaneous energy consumption calculation equation, and $\xi_{{2}}$ is the ability to quickly pass the traffic capacity of vehicles. It can be considered that the energy consumption minimum of AEVPs is important and that the quickly passing traffic capacity of vehicles cannot be accurately grasped. The weighting factor for $\mho_{1}$ can be set in the range of $\mho_{{1}} \in \left[ {{0}{\text{.6}}{0}{\text{.9}}} \right]$, and $\mho_{{2}} \in \left[ {{0}{\text{.1}}{0}{\text{.4}}} \right]$.

Equation (28) represents longitudinal vehicle dynamics, such as velocity and acceleration at time t of AEVPs. Equation (29) represents a longitudinal vehicle-following model.

When AEVPs are driving on road scenes that are prone to traffic accidents, the leading vehicle may drive at different energy-optimal speeds to ensure that the platoon can traverse traffic scenes safely. Therefore, the leading vehicle can be divided into two states at two different velocities. Equation (27) can be reformulated as an NLP as follows:

30 $$\mathop {\min }\limits_{{a_{1} ,a_{2} ,t_{1} ,t_{2} }} \xi = \mho_{1} \cdot \xi_{{1}} { + }\mho_{{2}} \cdot \xi_{{2}} { + }\mho_{{3}} \cdot \xi_{{3}} { = }\mho_{1} \cdot \sum\limits_{i} {\int_{{t_{0} }}^{{t_{1} }} {E_{i} \left( {v_{i} \left( t \right),a_{i} \left( t \right)} \right)} } dt + \mho_{{2}} \cdot \sum\limits_{i} {\int_{{t_{1} }}^{{t_{f} }} {E_{i} \left( {v_{i} \left( t \right),a_{i} \left( t \right)} \right)} dt} + \mho_{{3}} \cdot t_{f} \cdot$$

The objective function (30) is formulated in two steps: (i) the leader drives at constant acceleration or deceleration $a_{1}$ during time interval $\left[ {t_{0} ,t_{1} } \right)$, and (ii) the leader drives at constant acceleration or deceleration $a_{2}$ during time interval $\left[ {t_{1} ,t_{f} } \right)$. The constraint equations are equal to those mentioned above.

## Numerical experiments

### Experiment cases

This section describes numerical experiments to demonstrate the ecological driving of AEVPs. Two phases of experiments can be studied in this paper. Two obstacles on roads are stochastically set up and marked in red under the road environment. The obstacles can be expressed by using the P-norm as follows:

31 $$\left\| {\left( {x,y} \right)} \right\|_{p} = \left( {\left[ {\left| x \right|} \right]^{p} + \left[ {\left| y \right|} \right]^{p} } \right)^{1/p} ,p = 1,2, \ldots$$

When the value p is larger, the rectangular shape is closer to the vehicle. According to the above additions, the obstacle constraints can be set as follows:

32 $$h\left( {x,y} \right) = \log \left( {\left( {\frac{{x - x_{c} }}{a}} \right)^{p} + \left( {\frac{{y - y_{c} }}{b}} \right)^{p} } \right) \ge p \cdot \log c$$

where $\left( {x_{c} ,y_{c} } \right)$ is the center of obstacle. $\left( {a,b,c} \right)$ are the given parameters. The path constraints can be defined as follows:

33 $$h\left( {x,y} \right) \ge 0$$

The experiments can be divided into two phases, and the average traffic speed of AEVPs is assumed to be $10\,\text{m}{/}\text{s}$. In the first phase, suppose that the platoon consists of two vehicles, and some important parameters can be set up in the following form:

(i) The initial conditions for the leading vehicle and the following vehicle can be defined as follows:
  $$\begin{aligned} & x_{{{1 - }0}} = 0\,\text{m},y_{{{1 - }0}} = 0\,\text{m},v_{{{1 - }0}} = 10\,\text{m}{/}\text{s}\, \hfill \\ & s_{{1{ - }0}} = {12}\,\text{m},v_{{1{ - }0}} = 10\,\text{m}{/}\text{s} \hfill \\ \end{aligned}$$
(ii) The terminal conditions for the leading vehicle and the following vehicle can be defined as follows:
  $$\begin{aligned}& x_{{1 - f}} = 200\,\text{m},y_{{1 - f}} = - 2\,\text{m},v_{{1 - f}} = {2}0\,\text{m}{/}\text{s}, \hfill \\ & s_{{1{ - }f}} = {10}\,\text{m},v_{{1 -f}} = 1{0}\,\text{m}{/}\text{s} \hfill \\ \end{aligned}$$

Ecological driving study results for AEVPs are shown in the following graphics.

**Figure 2:**
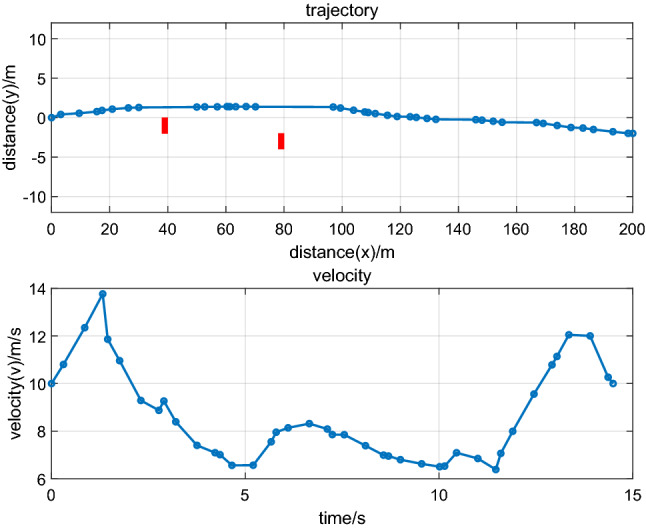
Trajectory optimization and driving states of the leading vehicle.

**Figure 3:**
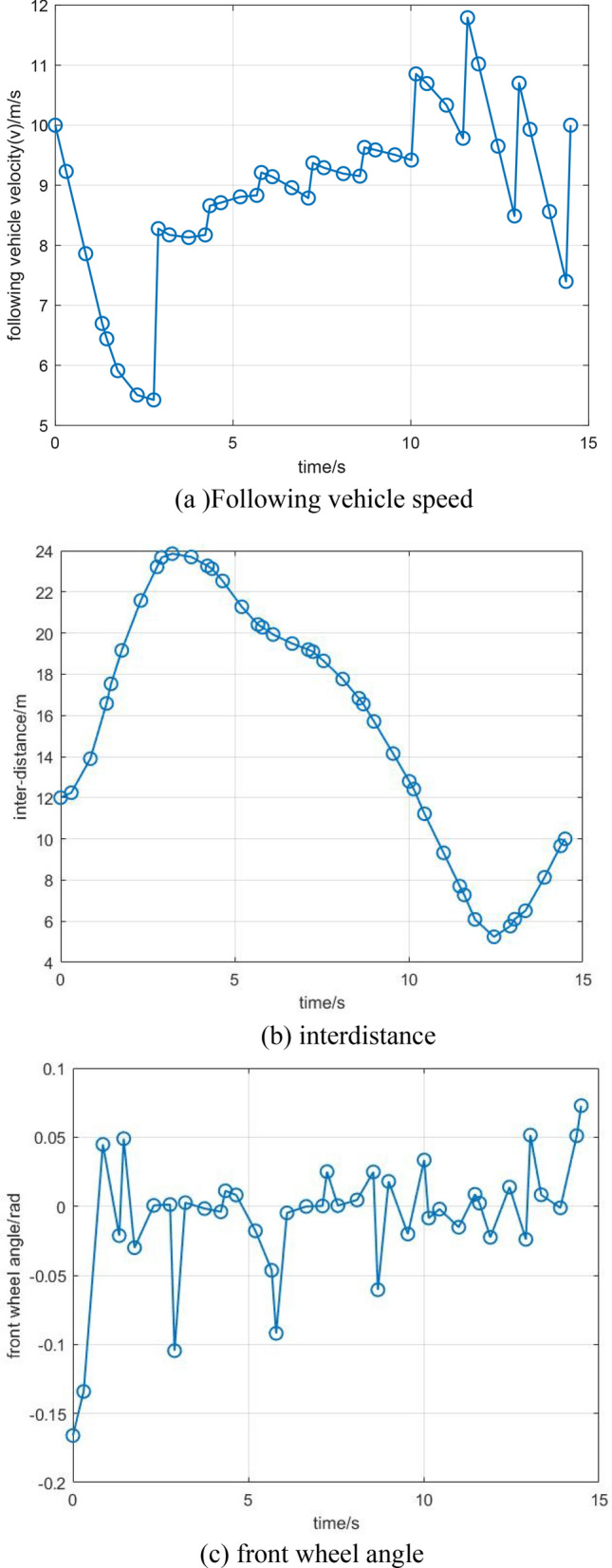
(**a**), (**b**), and (**c**) Driving states of the following vehicle.

Figure 2 illustrates the trajectory optimization results and driving states of the leading vehicle. As seen from Fig. 2, the planned trajectories can effectively avoid obstacles by considering the energy consumption minimum, vehicle dynamics, the ability to arrive at its direction as fast as possible, etc., which can be regarded as a general path for AEVPs by using RPM. Figure 3 illustrates the driving states of the following vehicle. It can be shown that driving states for the following vehicle tend to arrive at ecological forms by connecting vehicle technology. Using RPM to optimize the driving states of leading vehicles helps platoons realize ecological driving, thereby ensuring the goal of minimizing energy consumption.

In the second phase, the leading vehicle drives at an acceleration state, and some important parameters can be set up in the following form:

(i) The initial conditions for the leading vehicle and the following vehicle can be defined as follows:
  $$\begin{aligned} x_{{2 - 0}} &= 200\,\text{m},y_{{2 - 0}} = - 2\,\text{m}, \hfill \\ v_{{2 - 0}} &= 10\,\text{m}{/}\text{s}, \,\,\text{s}_{{1{ - 0}}} = {10}\,\text{m},v_{{1{ - 0}}} = 10\,\text{m}{/}\text{s} \hfill \\ \end{aligned}$$
(ii) The terminal conditions for the leading vehicle and the following vehicle can be defined as follows:
  $$\begin{aligned} x_{{2 - \text{f}}} &= 400\,\text{m},y_{{2 - \text{f}}} = 0\,\text{m}, \hfill \\ v_{{{2 - \text{f}}}} &= 20\,\text{m}{/}\text{s,} \,\,\text{s}_{{1{{ - \text{f}}}}} = 6\,\text{m},v_{{1{{ - \text{f}}}}} = 20\,\text{m}{/}\text{s} \hfill \\ \end{aligned}$$

**Figure 4:**
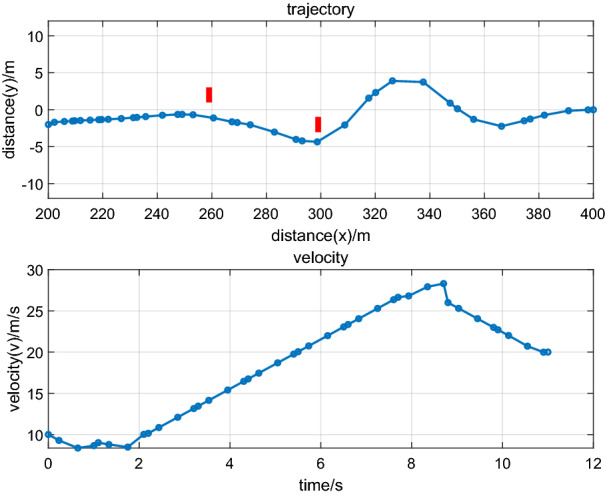
Trajectory optimization and driving state of the leading vehicle.

**Figure 5:**
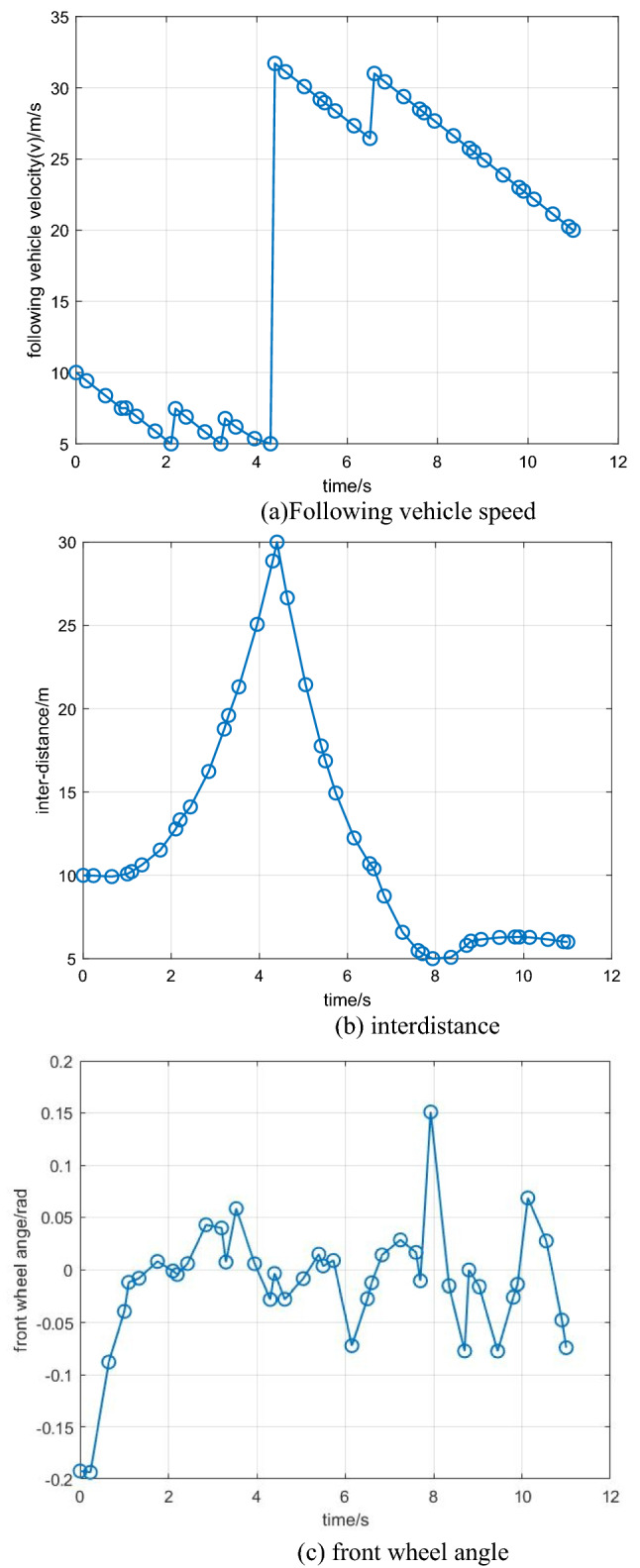
(**a**), (**b**), and (**c**) Driving states of the following vehicle.

Figure 4 illustrates trajectory optimization and driving states of the leading vehicle that accelerate in the second phase. An effective trajectory is designed by using RPM with position changes of obstacles. As shown in Figs. 4 and 5, the platoon can regulate driving states to realize ecological driving for AEVPs by using RPM to design the states of the leading vehicle. The results show that AEVPs can automatically adjust driving states when terminal conditions and discrete control variables and input variables in multiple phases are taken into consideration, while the energy consumption of all electric vehicles can be designed, thereby realizing ecological driving for AEVPs.

Suppose that the platoon consists of three vehicles, and the average traffic speed of AEVPs is assumed $10\,\text{m}{/}\text{s}$ to be to explain the ecological driving process. The driving states for AEVPs are studied in the first phase as follows:

(i) The initial conditions for the leading vehicle and the following vehicles can be defined as follows:
  $$\begin{aligned} x_{{1 - 0}} &= {1}0\text{m},y_{{{1 - 0}}} = 0\text{m},v_{{{1 - }0}} = 10\,\text{m}{/}\text{s},\, \text{s}_{{1 - 0}} = {12}\,\text{m}, \hfill \\ v_{{1 - 0}} & = 10\,\text{m}{/}\text{s}, \,\,\text{s}_{{1 - 0}} = {12}\,\text{m},v_{{1- 0}} = 10\,\text{m}{/}\text{s} \hfill \\ \end{aligned}$$
(ii) The terminal conditions for the leading vehicle and the following vehicles can be defined as follows:
  $$\begin{aligned} x_{{1 -f}} &= 200\,\text{m},y_{{1 - f}} = - 2\,\text{m},\,v_{{{1 - }f}} = 10\,\text{m}{/}\text{s}, \hfill \\ \text{s}_{{1- \text{f}}} &= {10}\,\text{m},\,\,v_{{1 -f}} = 10\,\text{m}{/}\text{s}, \,\,\text{s}_{{1 -f}} = {10}\,\,\text{m},v_{{1 -f}} = 10\,\text{m}{/}\text{s} \hfill \\ \end{aligned}$$
  In the second phase, the initial conditions for the leading vehicle and the following vehicle are equal to the terminal conditions in the first phase.
(iii) The terminal conditions can be defined as follows:
  $$\begin{aligned} x_{{2 - f}} &= 400\;{\text{m}},y_{{{1 - }f}} = - 2\;{\text{m}},v_{{2{ - }f}} = 10\;{\text{m/s}}, \hfill \\ \text{s}_{{{2 - }f}} &= {10}\;{\text{m}},v_{{2{ - }f}} = 10\;{\text{m/s}}, \,\,\text{s}_{{2{ - }f}} = {10}\;{\text{m}},v_{{2{ - }f}} = 10\;{\text{m/s}} \hfill \\ \end{aligned}$$
  The ecological driving study results for AEVPs are shown in the following graphics.

**Figure 6:**
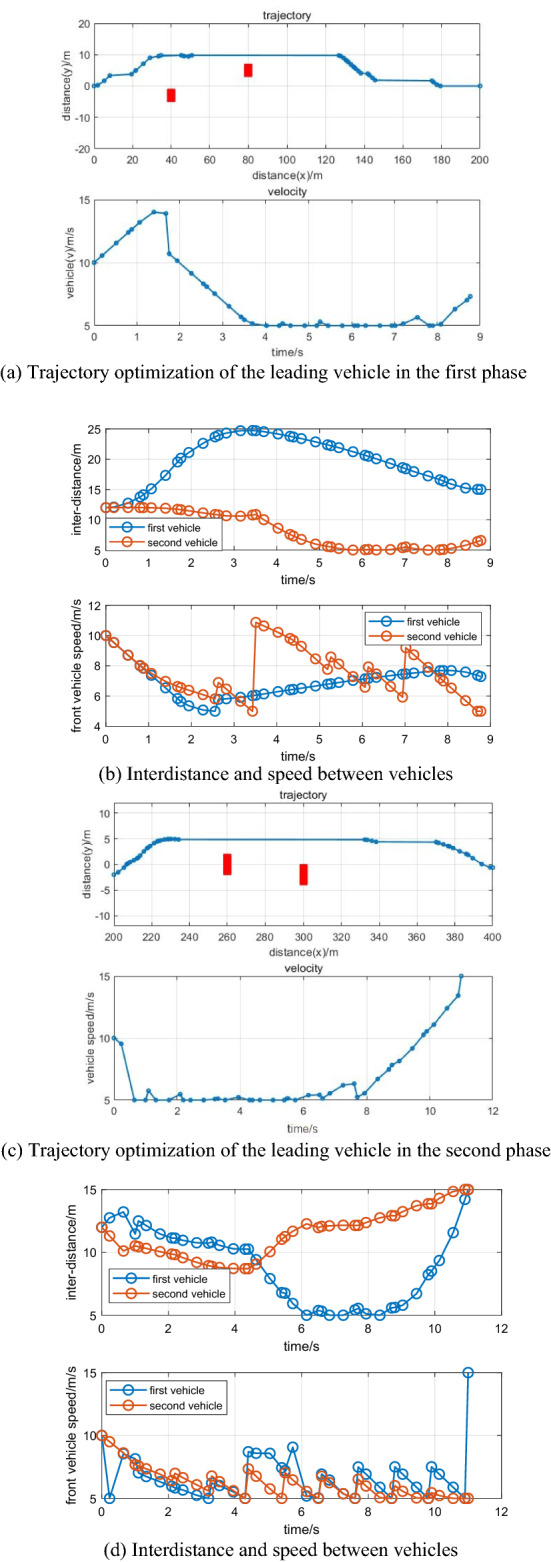
(**a**–**d**) The driving states of AEVPs in two driving phases.

Figure 6 illustrates trajectory optimization and driving states of the leading vehicle. Figure 6 shows the driving states of the following vehicles. When the trajectory and driving states of the leading vehicle are developed by RPM, vehicle states can be transmitted to the following vehicles by V2 V technologies to effectively track the planned trajectory and driving states. Meanwhile, the minimum total energy consumption can be optimized by the optimal model. Figure 6 shows that the following vehicles can change driving states with the leading vehicle states. Obstacle position avoidance can influence trajectory planning and driving state constraints of the leading vehicle, thereby influencing the following vehicle driving states and interdistances. Usually, the leading vehicle tends to reduce platoon states to optimize overall energy consumption, so RPM is adopted to optimize and design driving states of the leading vehicle to expediently calculate total platoons of driving states. The greatest advantage in AEVPs is that the automated leader can adjust its driving states according to a specific traffic environment, allowing other following vehicles to adjust their driving states smoothly, thereby minimizing total energy consumption and realizing ecological driving for AEVPs.

### Comparative analysis

**Figure 7:**
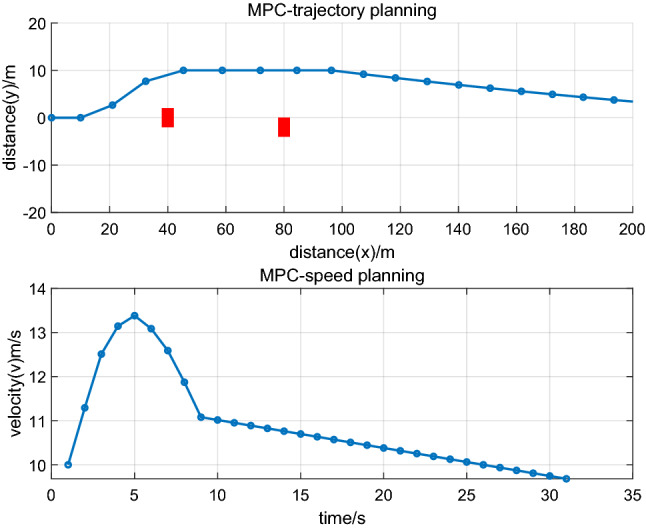
trajectory optimization of vehicle.

**Figure 8:**
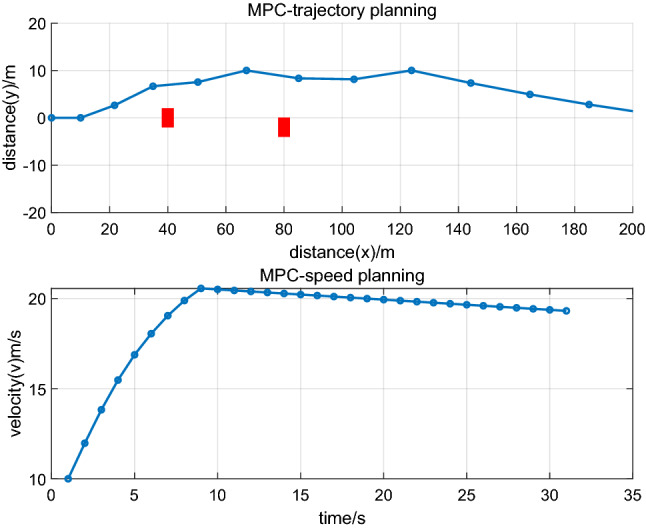
trajectory optimization of vehicle.

This section describes a comparative analysis of RPM in realizing multiphase trajectories and multiobjective optimization for AEVPs. As trajectory planning is based on the optimal control method, the model predictive control method (MPC) is another trajectory planning method for autonomous electric vehicles. To reflect the advantage of the RPM method, the comparative analysis is studied. Trajectory planning based on MPC and vehicle velocity are set as $10\;{\text{m/s}}$ and ${2}0\;{\text{m/s}}$, respectively. The simulation process is as Figs. 7 and 8:

Figure 7 illustrates trajectory optimization and driving states of the leading vehicle under a velocity of $10\;{\text{m/s}}$, and Fig. 8 illustrates trajectory optimization and driving states of the leading vehicle under a velocity of ${2}0\;{\text{m/s}}$. From Figs. 7 and 8, it can be seen that under different speeds, vehicles can realize obstacle avoidance. Compared with Figs. 2, 4 and 6, it can also be seen that the discrete points based on the RPM algorithm are uneven, which is used to design trajectory planning of autonomous vehicles. It can scatter more dense discrete points when there are some obstacle positions, and the discrete points are loose when the distance between vehicles and obstacles is far. The process can effectively realize the obstacle avoidance function and realize global optimization under the process of trajectory planning of autonomous vehicles. The discrete points based on the MPC algorithm are uniform and are used to design trajectory planning of autonomous vehicles. The discrete points are of identical density regardless of whether there are obstacles, which can cause the solution results to tend to fall into a local optimum and cannot ensure trajectory planning of global optimization. Therefore, the RPM algorithm is used to study the ecological driving of AEVPs and contributes to trajectory planning of global optimization. It has the advantage of multiphase trajectory planning where the energy consumption minimum is considered, the driving states of the leading vehicle are optimized according to traffic scenes, and the total energy consumption minimum of the platoon is finally realized.

## Conclusions

AEVPs is a major research hotspot in future. This paper proposes an optimal control model for ecological driving to minimize the energy consumption of AEVPs. To enhance ecological driving efficiency, the proposed optimal control model develops NLP problems by using the RPM algorithm. NLP problems easily solve such propositions because they contain some decision variables and constraints. By solving the NLP formulation, the ecological driving states and trajectory of the leading vehicle can be obtained, and a sequence of driving states, such as acceleration and velocity, can be suggested to the following vehicles for automated vehicle platoons. Numerical experiments illustrate ecological driving strategies for AEVPs and demonstrate the benefits of connected and autonomous vehicle technologies in realizing energy efficiency and ecological driving of platoons. Numerical experiments also show that regarding energy consumption efficiency, connected and autonomous vehicle systems with platoons can effectively realize total energy consumption efficiency. Meanwhile, the multiphase trajectories of autonomous vehicles by the RPM algorithm can be further investigated during some complicated traffic scenes through complex communication topology. For example, when AEVPs drive at unsignalized intersections, so many autonomous vehicles come from different directions. To ensure that AEVPs safely drive, the complex communication topology can be used to transfer vehicle state information to the surrounding vehicles to plan multiphase trajectory planning, which can be helpful to realize specific driving states during different driving phases. Combined with complex communication topology, multiphase trajectory planning by the RPM method can be used to solve so many complicated road traffic scenes, such as tunnel traffic scenes, road traffic in severe weather, and bridge traffic that has the features of wet and slippery roads.
